# Supplementary material for: Direct observation of cation diffusion driven surface reconstruction at van der Waals gaps
Source: Nat Commun. 2023 Feb 2;14:554. doi: 10.1038/s41467-023-35972-9 (PMC9894939; doi:10.1038/s41467-023-35972-9)
Supplement: Supplementary file 1 — Supplementary information [file 41467_2023_35972_MOESM1_ESM.pdf]

Supplementary Information for

Direct observation of cation diffusion driven surface reconstruction at van der Waals gaps

Wenjun Cui et al.

This Supplementary Information includes:

I. Supplementary Figures 1-23

II. Supplementary Tables 1-3

III. Supplementary References

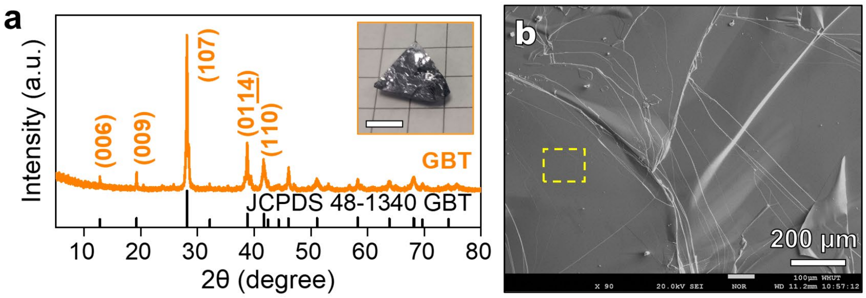

Supplementary Figure 1 | X ray diffraction (XRD) and scanning electron microscopy (SEM) characterization of as-prepared $GeBi_2Te_4$ (GBT) bulk material. (a) The experimental diffraction peaks agree well with the hexagonal phase GBT (JCPDS#48-1340, $R\bar{3}m$ space group). The inset is a picture of the GBT ingot with scale bar 5 mm. (b) SEM image of the exfoliated surface of GBT bulk material, the yellow box shows the area where FIB samples were prepared.

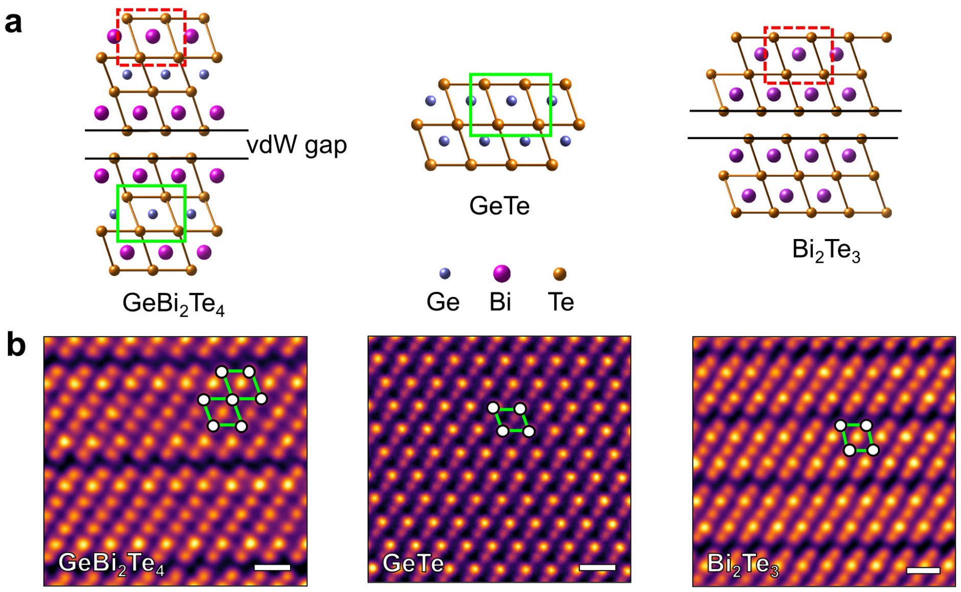

Supplementary Figure 2 | Crystal structure and experimental STEM images of GBT, GeTe and Bi_2Te_3 structure along the [$\bar{2}$110] zone axis. (a) Projected atomic structure models of GBT, GeTe and Bi_2Te_3. Red dashed and green solid boxes indicate Bi occupied and Ge occupied Te_6 octahedrons. (b) Atomic resolution annular dark-field scanning transmission electron microscopy (ADF-STEM) images of GBT, GeTe and Bi_2Te_3 along the [$\bar{2}$110] zone axis. All theoretical and experimental (α, h) values of the different octahedrons are shown in Supplementary Table 1[1-3]. All scale bars are 0.5 nm.

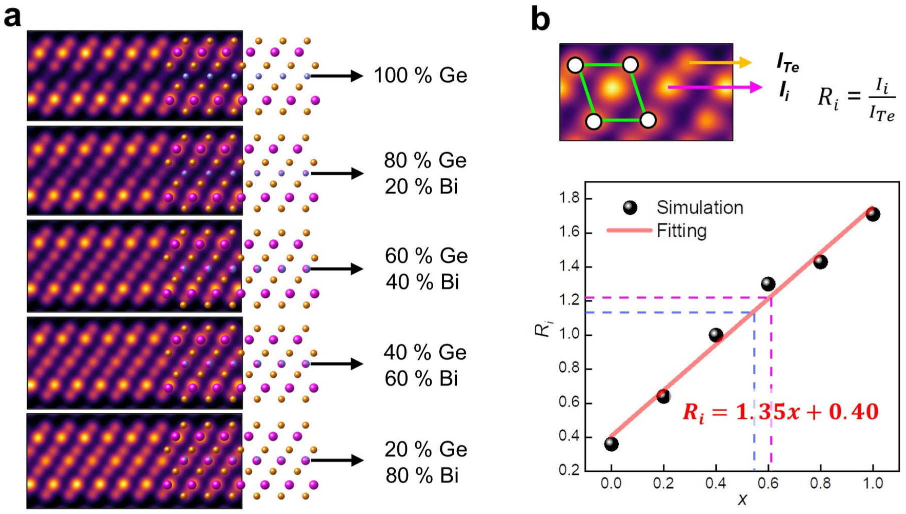

Supplementary Figure 3 | Relationship of the Bi cation concentration in the Ge atomic column and the STEM images intensity. (a) Simulated ADF-STEM images with 0%, 20%, 40%, 60% and 80% Bi concentration in the Ge atomic columns. (b) To eliminate the thickness influence[4], the normalized STEM intensity R_i is defined as $R_i = \frac{I_i}{I_{Te}}$, where I_i and I_{Te} are the intensity of Ge/Bi atomic column and the average intensity of four adjacent Te columns. A linear relationship $R_i = kx + b$ between R_i and Bi concentration x was obtained with slope $k = 1.35 \pm 0.08$ and $b = 0.40 \pm 0.05$.

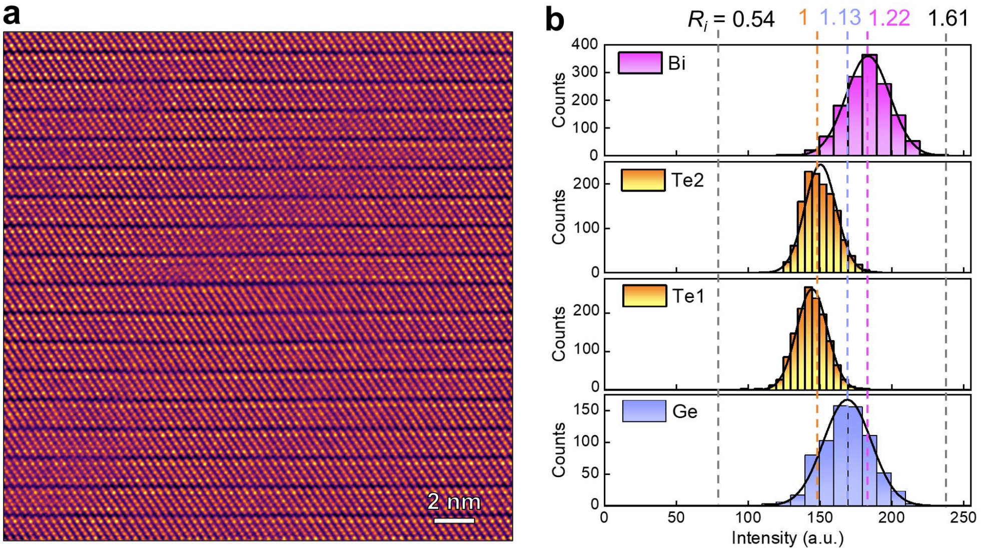

Supplementary Figure 4 | Quantitative analysis of Ge/Bi antisite defects in GBT crystals[5, 6]. (a) Atomic resolution ADF-STEM image of cross-sectional GBT was used for atomic columns statistical intensity analysis. (b) Histogram of the Ge, Bi and Te atomic columns intensity from (a). The average R_i of Ge and Bi atomic columns are 1.13 and 1.22, corresponding to 54% and 61% Bi concentration by simulation, respectively.

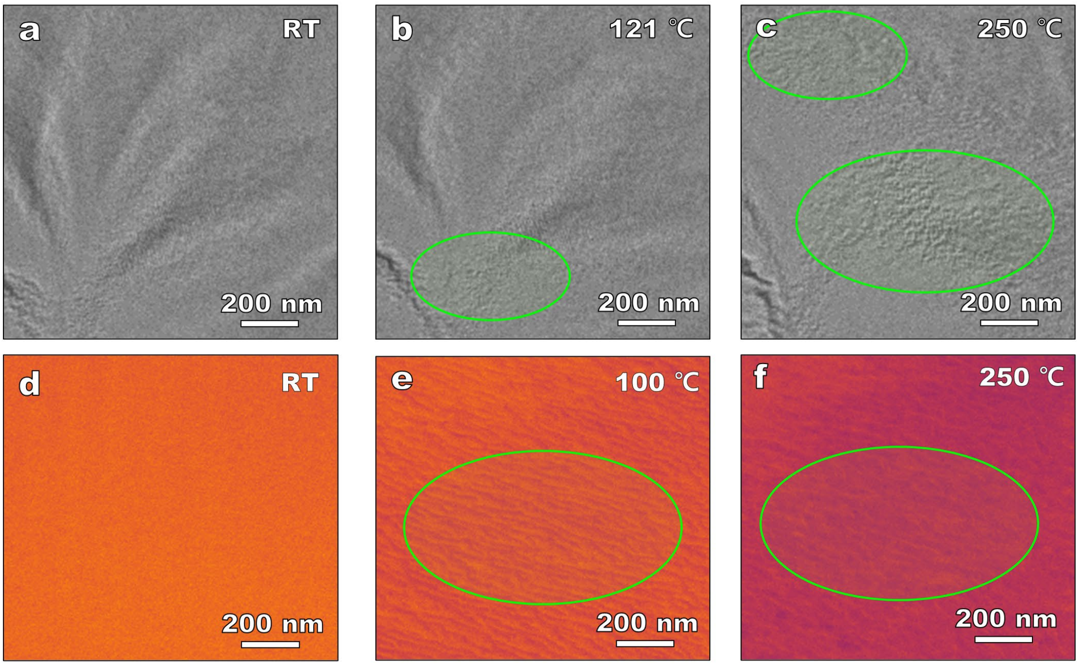

Supplementary Figure 5 | Low magnification *in situ* TEM images (a-c) and STEM images (d-f) during heating from room temperature (RT) to 250 °C. Stacking faults appear at regions indicated by green ovals.

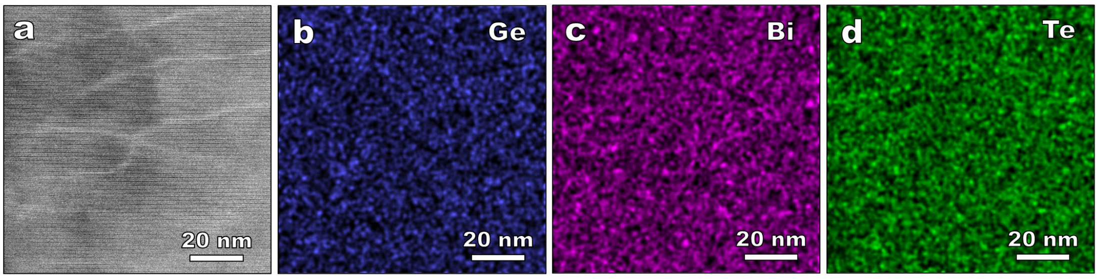

Supplementary Figure 6 | EDS element maps of Ge, Bi and Te acquired from the stacking fault region. (a) The ADF-STEM image shows the bright contrast corresponding to stacking faults. (b-d) EDS maps acquired from the region in (a). The Ge, Bi and Te signals are uniform across the region, proving that there is no noticeable segregation of cations or anions at the stacking fault.

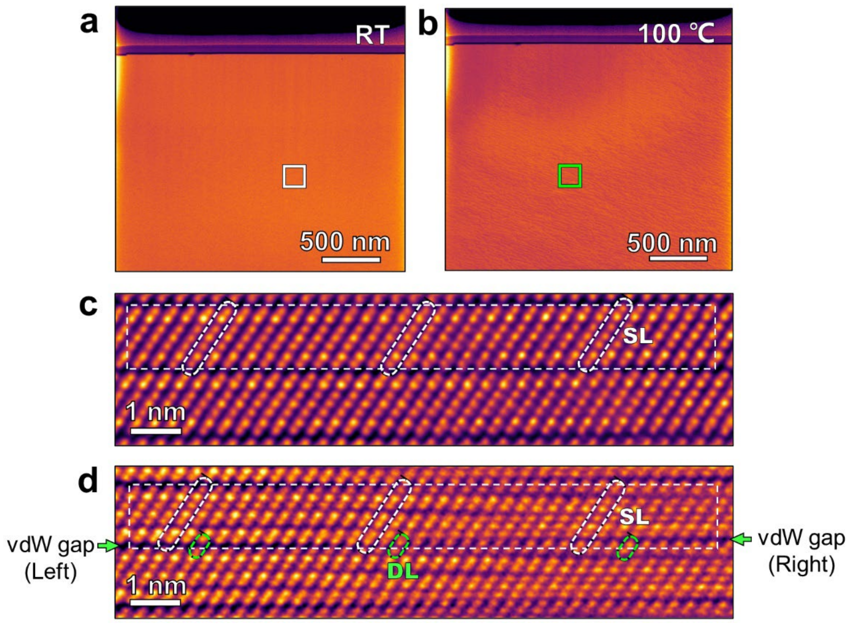

Supplementary Figure 7 | ADF-STEM images acquired from the layered defects area. Low magnification ADF-STEM image at RT (a) and 100 °C (b). (c, d) The corresponding atomic-resolved ADF-STEM images from the boxes in (a) and (b), respectively. We can see that the left vdW gap and right vdW gap are misaligned by one atomic layer at the stacking fault core region, where the vdW gap seems to be filled by extra atoms. Careful analysis however shows that this is visual effect, and there is no additional sublayer from cations going into vdW gaps. For example, the distance between the two Te sublayers at the left vdW gap gradually decreases from left to right, and then eventually form a double layer (DL) structure with a vdW gap above it (green rods and arrows).

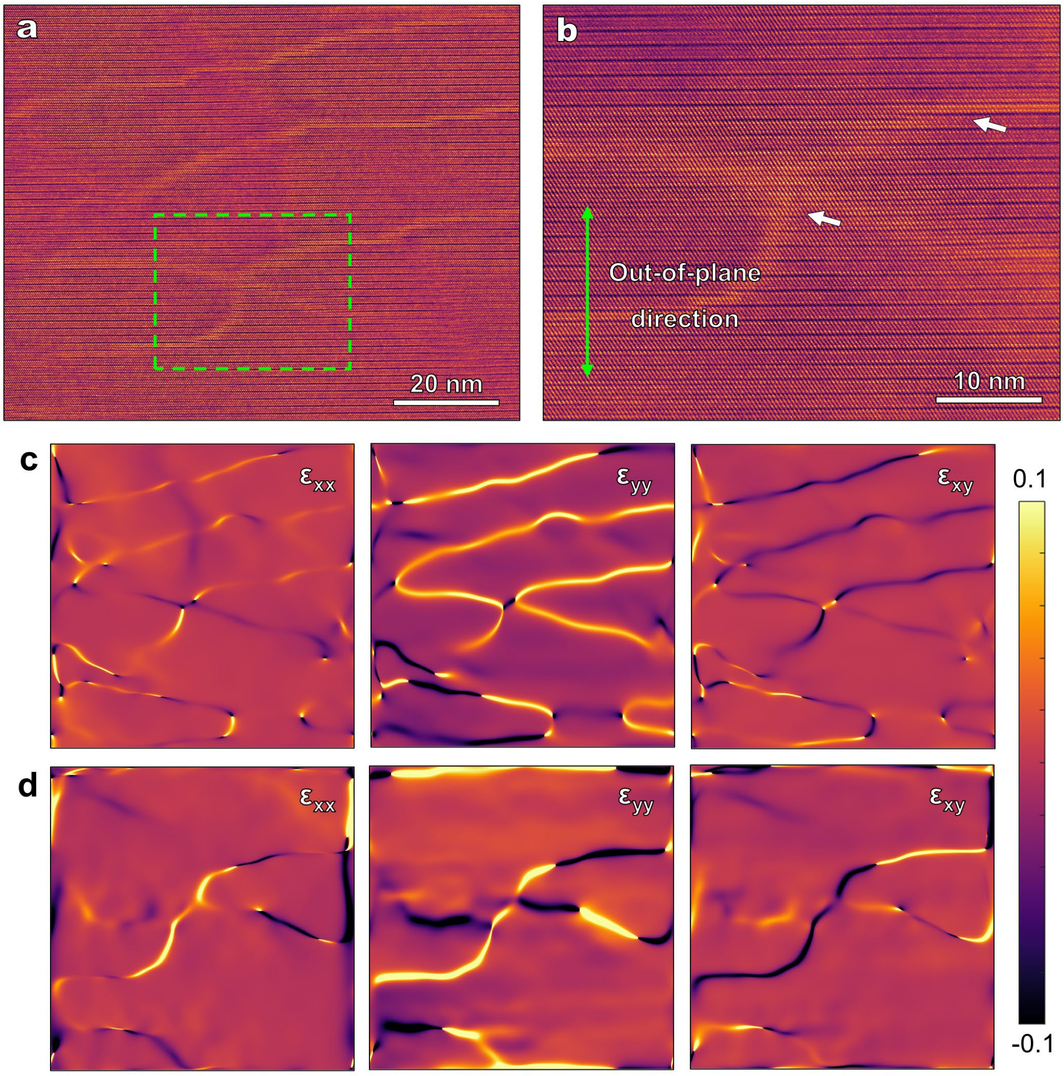

Supplementary Figure 8 | Geometric phase analysis (GPA) maps of the STEM images with mixed atomic DL, triple layer (TL), quintuple layer (QL) and septuple layer (SL) defect structures at 100 °C. (a) ADF-STEM image acquired from GBT at 100 °C. (b) Enlarged image of the green dashed line box in (a). The regions with brighter contrast coincide with mixed layer defects (indicated by white arrows). (c, d) GPA strain maps from (a) and (b), respectively. The strain originates from the formation of mixed layer structures[7, 8].

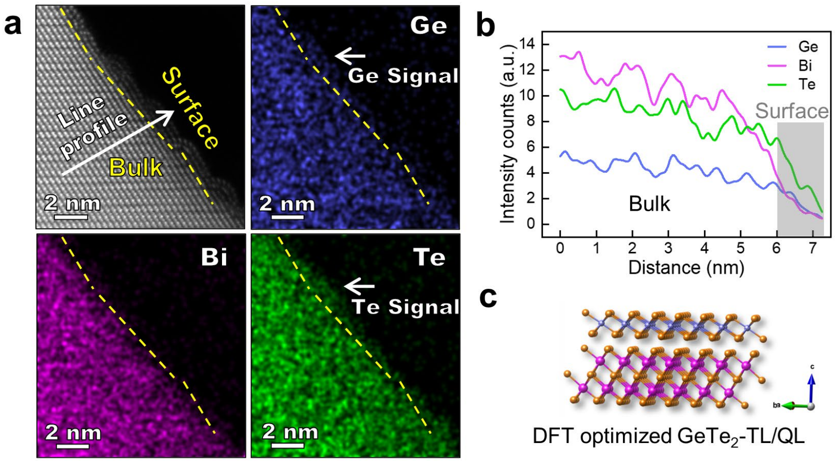

Supplementary Figure 9 | The composition and crystal structure of reconstructed $GeTe_2$ TL surface revealed by EDS and density functional theory (DFT). (a) EDS maps of Ge, Bi and Te element at a reconstructed $(01\overline{1}\overline{7})$ surface, with the yellow dashed line marked the interface between GBT and the $GeTe_2$ TL surface. (b) The line profile of EDS intensity along the white line in ADF-STEM image (a). (c) The interface structure between $GeTe_2$ TL and GBT QL was relaxed using DFT. The relaxed structure matches well with the experimental STEM image as shown in Fig. 1f.

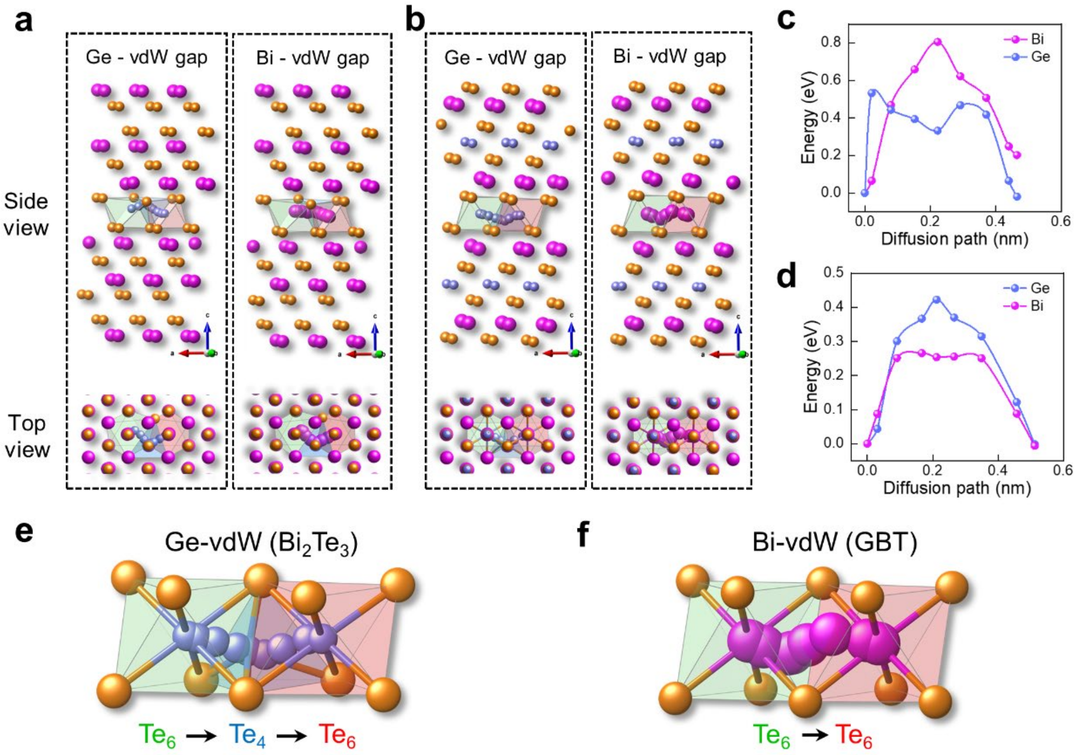

Supplementary Figure 10 | The diffusion energy barrier of Ge and Bi atoms in vdW gaps of Bi_2Te_3 and $GeBi_2Te_4$. (a, b) The migration path of Ge and Bi atoms diffusing along the vdW gaps of Bi_2Te_3 (a) and $GeBi_2Te_4$ (b) layered structure respectively. The calculation was performed using the climbing image nudged elastic band method (CI-NEB)[9] implemented in VASP 3×2×1 supercells of Bi_2Te_3 and $GeBi_2Te_4$ were used in the calculation The green and red octahedrons indicate initial-state and final-state octahedrons. The energy barriers of Ge and Bi cation diffusion in vdW gap of Bi_2Te_3 and $GeBi_2Te_4$ are plotted in (c) and (d), respectively. (e) The diffusion path of Ge cation in Bi_2Te_3 vdW gap. The Ge cation first diffuses into the adjacent blue tetrahedron and then the red octahedron. (f) The diffusion path of Bi cation in GBT vdW gap. The Bi cation directly diffuses from the initial green Te_6 octahedron to the adjacent red Te_6 octahedron without entering the Te_4 tetrahedron.

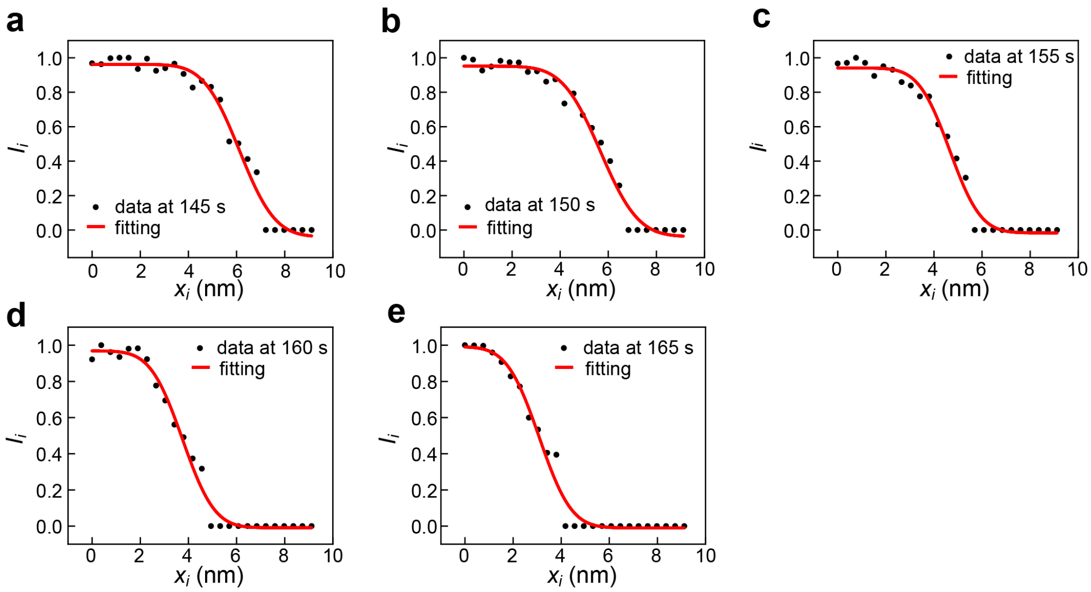

Supplementary Figure 11 | Curve fitting of the cation concentration I_i and distance x_i by Fick's law. (a-e) The fitting results on cation concentration I_i and distance x_i in Fig. 2d at 145 *s*, 150 *s*, 155 *s*, 160 *s* and 165 *s* using Fick' law.

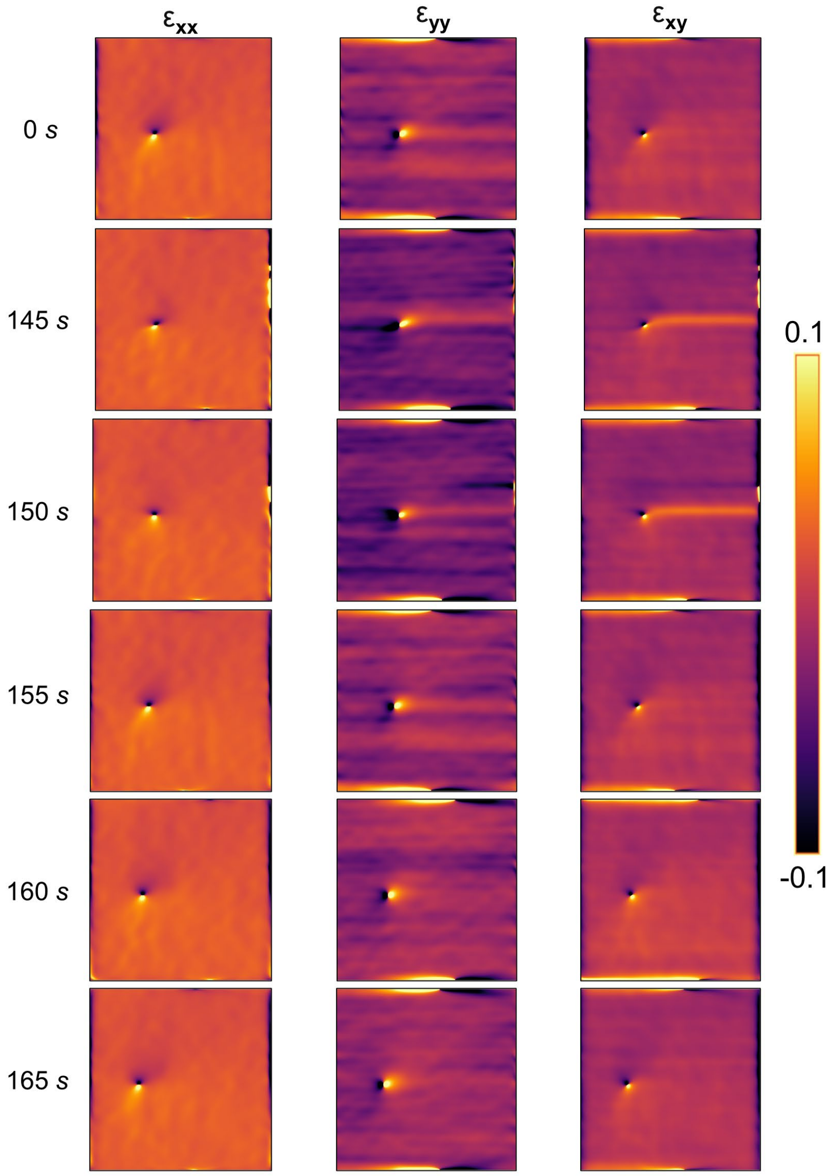

Supplementary Figure 12 | *In situ* GPA maps during cation diffusion along the van der Waals gap. The GPA maps of aligned STEM images at 0 *s*, 145 *s*, 150 *s*, 155 *s*, 160 *s* and 165 *s*, extracted from Supplementary Movie 2. The (0006) and ($01\bar{1}\bar{7}$) diffraction spots were selected to calculate the GPA maps.

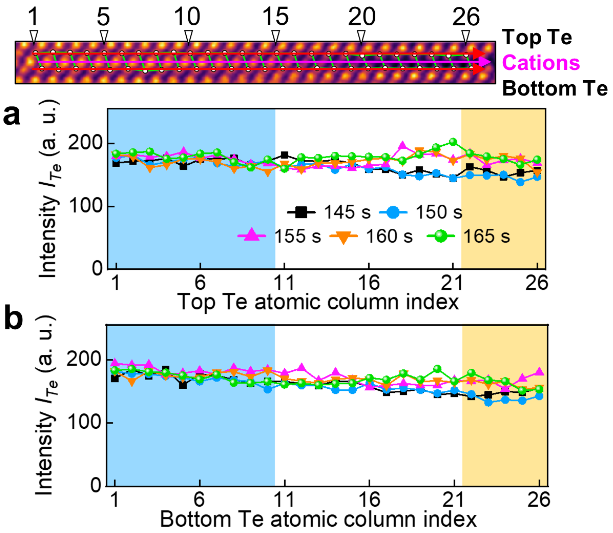

Supplementary Figure 13 | Intensity I_{Te} of the atomic columns in top Te, bottom Te sublayers. (a, b) Line intensity profile of top Te and bottom Te atomic columns. Blue and yellow shades indicate fully occupied and unoccupied areas at 145 *s*, respectively. There is no obvious variation from the 1st to the 26th Te atomic column, which confirms that the Te anionic columns are stable during cation diffusion.

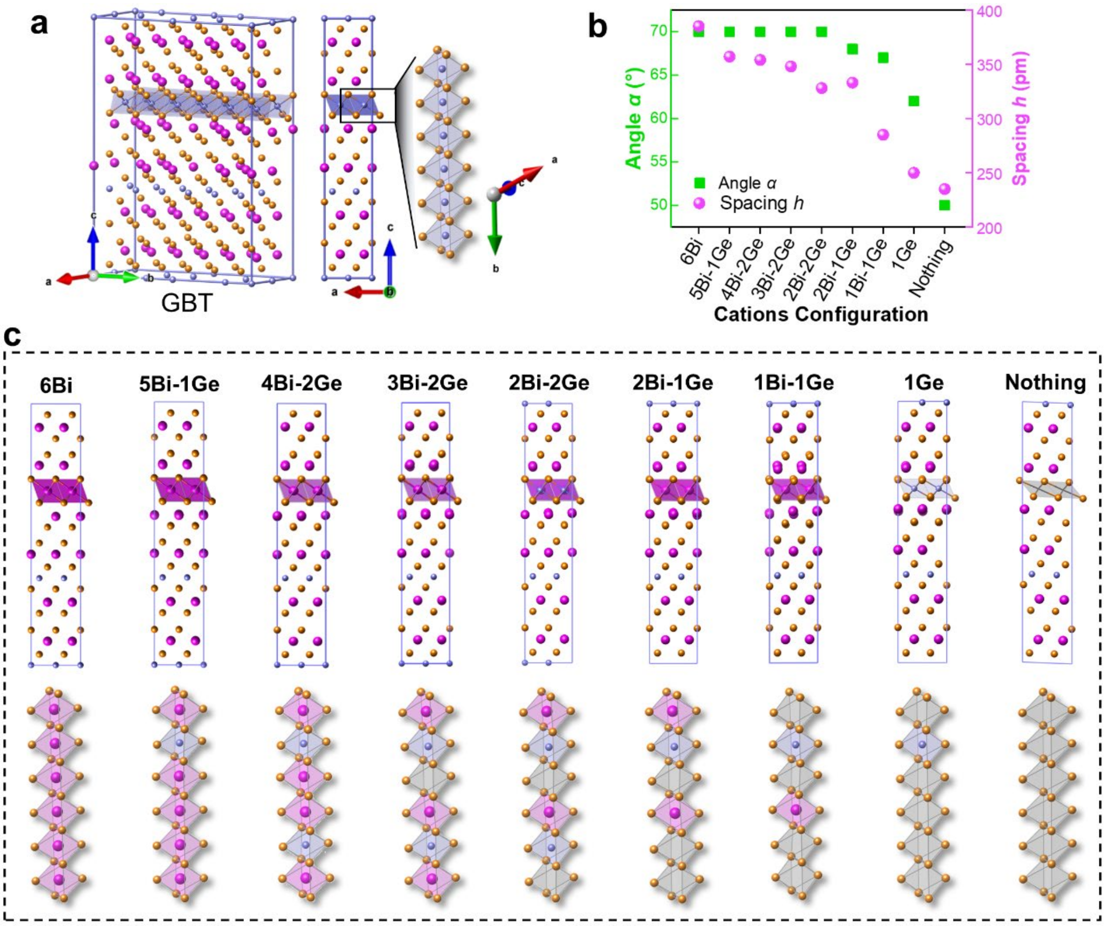

Supplementary Figure 14 | DFT calculation showing how the average geometric parameters of the projected Te_6 octahedrons are influenced by cation concentration of the atomic column. (a) The atomic structure model of a 2×6×1 GBT supercell. When projected along the *b* axis, the STEM image comes from the overlap of six Te_6 octahedrons. We now focus on the octahedron framed by the black square. The arrangement of this octahedron along *b* axis is shown next to the black square. (b) The (α, h) parameters for different combination of cations along the *b* axis, including 6 Bi cations, 5 Bi and 1 Ge cations, 4 Bi and 2 Ge cations, 3 Bi and 2 Ge cations, 2 Bi and 2 Ge cations, 2 Bi and 1 Ge cations, 1 Bi and 1 Ge cations, 1 Ge cation, and no cation occupancy. Both the spacing and the angle changes abruptly when the cation concentration is around 50%. (c) The crystal structure models of GBT supercells with different cation concentration.

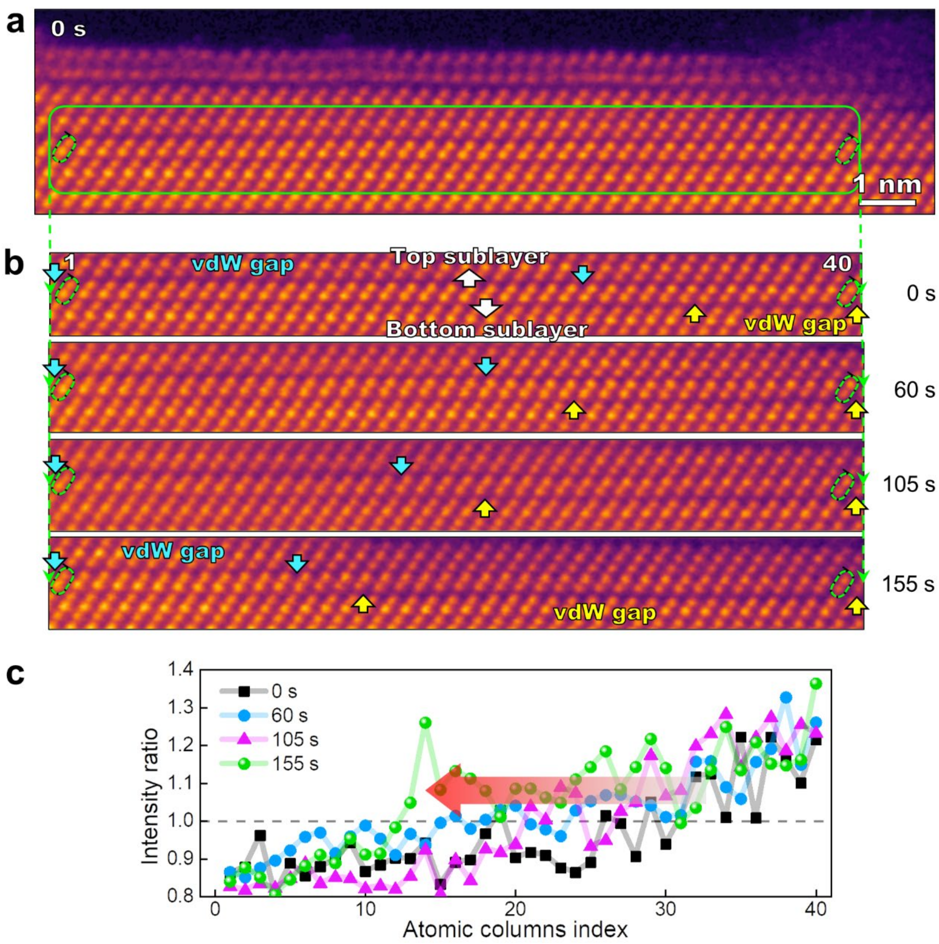

Supplementary Figure 15 | The intensity ratio of atomic column pairs in DL structure from Fig. 4, for frames acquired at different time stamp. (a, b) Atomic resolution ADF-STEM images acquired at 0 *s*, 60 *s*, 105 *s* and 155 *s*. The specific DL for intensity ratio analysis is marked by a green box in each image. The white arrows indicated top and bottom sublayers. The atomic column pair (marked by green rods) has one atomic column from the top sublayer and another atomic column from the bottom sublayer. Cyan and yellow arrows corresponding to the vdW gaps above the top sublayer and below the bottom sublayer. (c) The I_{top}/I_{bottom} intensity ratio for every atom pair in the DL structure from left to right for different frames from 0 *s* to 155 *s*. For completely disordered structure, the ratio should be close to 1. If the top layer is rich in Bi/Ge, the ratio is larger than 1; if the top layer is rich in Te, then the ratio is less than 1. For each frame, the ratio increases from left to right and crosses the ratio = 1 line. When the intensity ratio is less than 1, the vdW gap tends to appear above the top sublayer. When the intensity ratio is larger than 1, the vdW gap tends to appear

below the bottom sublayer. As time evolves, the crossover position moves to the left as indicated by the red arrow, suggesting that cations gradually diffuse to the top layer. The vdW gap above the top layer gradually closes, forming the QL structure.

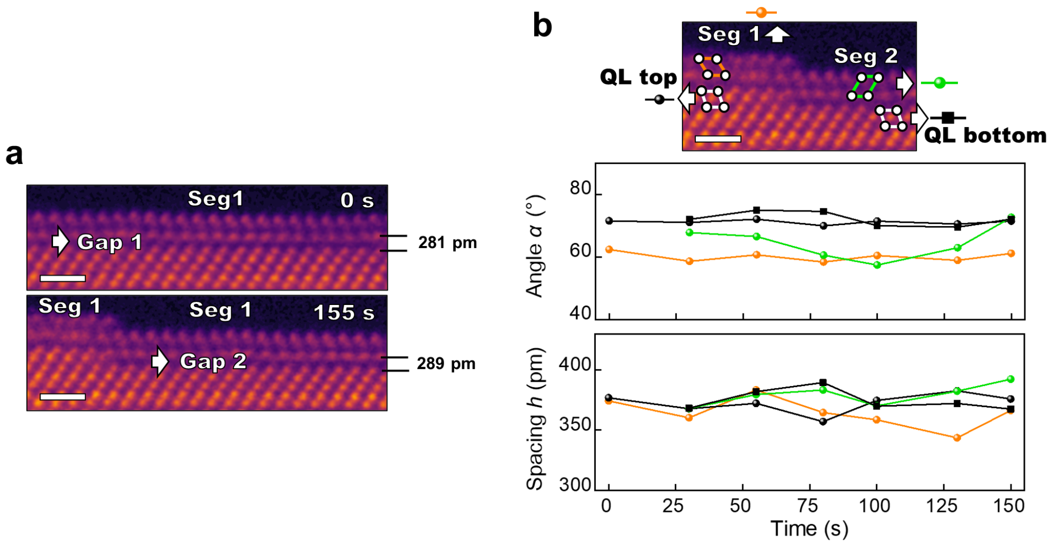

Supplementary Figure 16 | Quantitative analysis of Te_6 octahedrons during (0001) surface reconstruction. (a) The spacing *h* of Gap 1 and Gap 2 are 281 pm and 289 pm, respectively. (b) Quantitively results (*α*, *h*) of octahedrons in Segment 1 (Seg 1), Seg 2, QL top and bottom at 0 *s*, 30 *s*, 55 *s*, 75 *s*, 100 *s*, 130 *s* and 150 *s*. All scale bars are 1 nm.

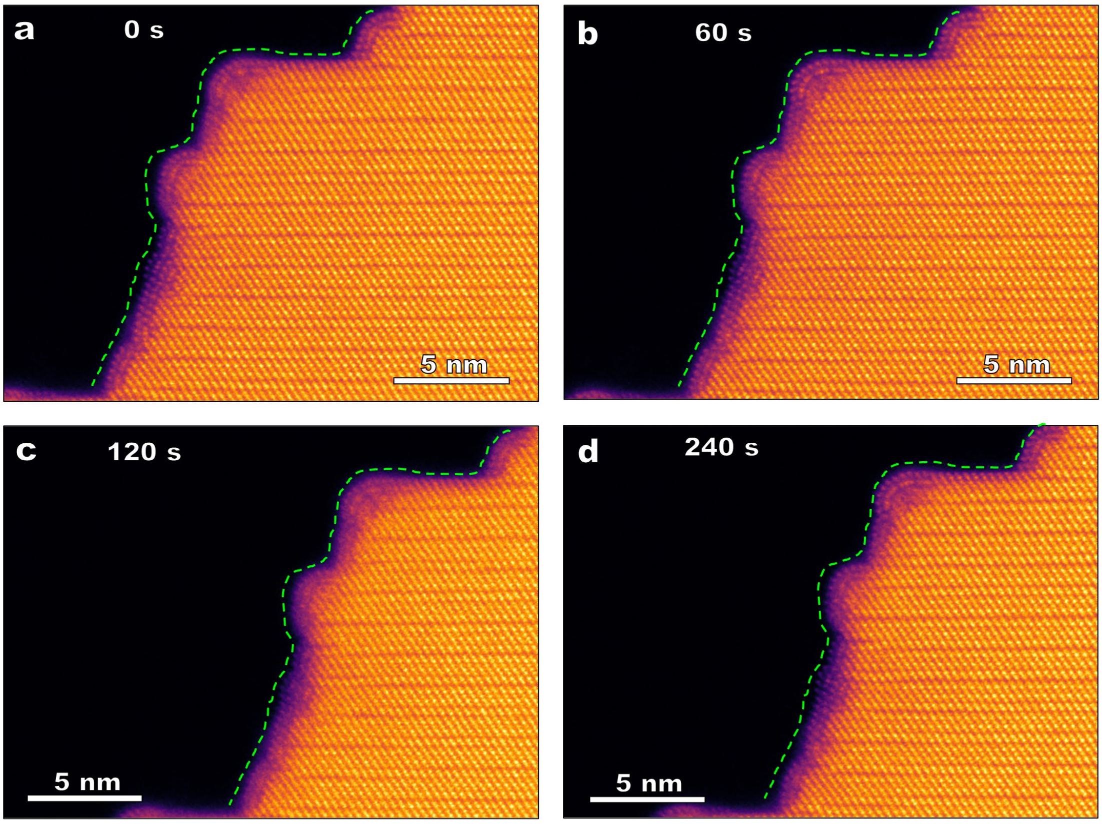

Supplementary Figure 17 | *In situ* ADF-STEM frames showing that the reconstructed surface was stable under e-beam irradiation when the temperature was kept at 150 °C. (a) ADF-STEM image of the reconstructed surface at 400 °C. (b-d) The reconstructed surface under e-beam irradiation after 60 *s* (b), 120 *s* (c) and 240 *s* (d) when the temperature was kept at 150 °C. The green dashed lines indicate the outline of reconstructed surface in (a-d). The surface construction does not change under beam irradiation at 150 °C. This proves that the reconstruction is only activated above certain temperature.

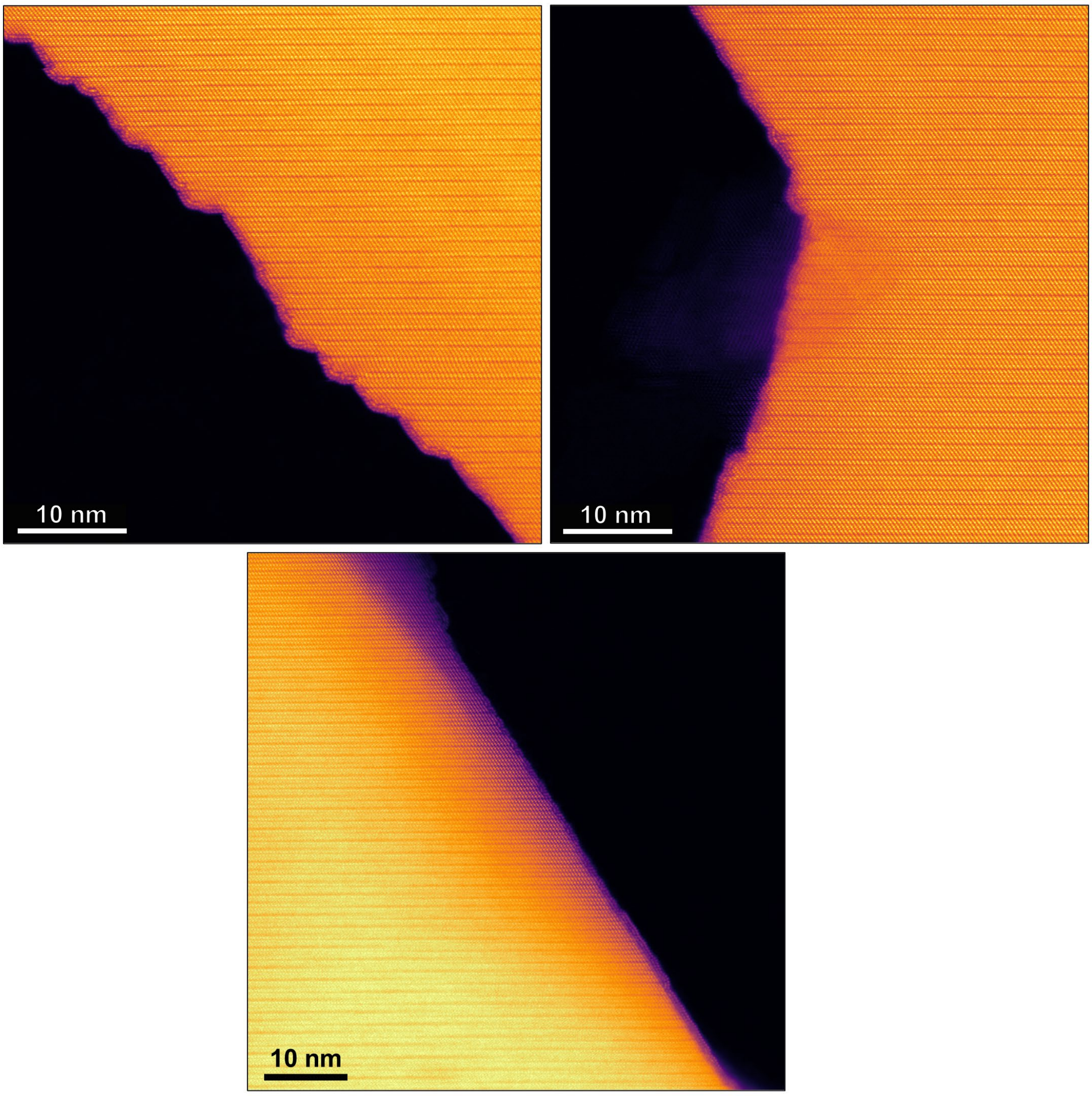

Supplementary Figure 18 | Formation of $GeTe_2$ TL reconstructed surfaces without electron beam irradiation. These STEM images were acquired by quickly moving to regions that have not been beam irradiated.

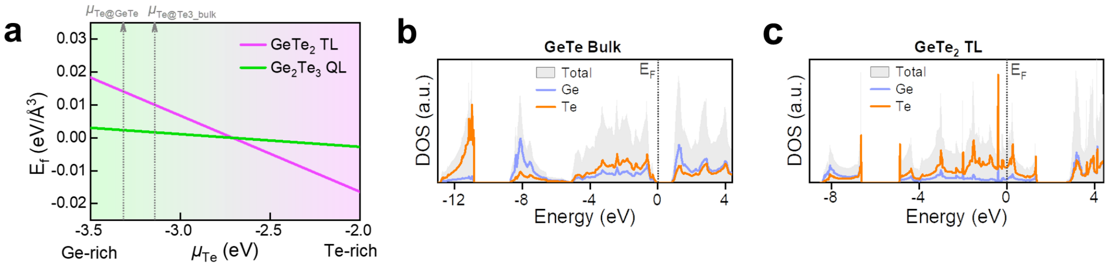

Supplementary Figure 19 | Formation energies and the density of state of $GeTe_2$ TL surface reconstruction calculated using DFT. (a) Formation energies (E_f) of the $GeTe_2$ TL and Ge_2Te_3 QL as a function of Te chemical potential (μ_{Te}). Here, μ_{Te} in Te_3 bulk ($\mu_{Te@Te3\_bulk}$ = -3.142 eV) is set as reference energy. (b, c) Density of state (DOS) of bulk GeTe (b) and $GeTe_2$ TL (c). The GeTe bulk material is semiconductor while the $GeTe_2$ TL is metallic.

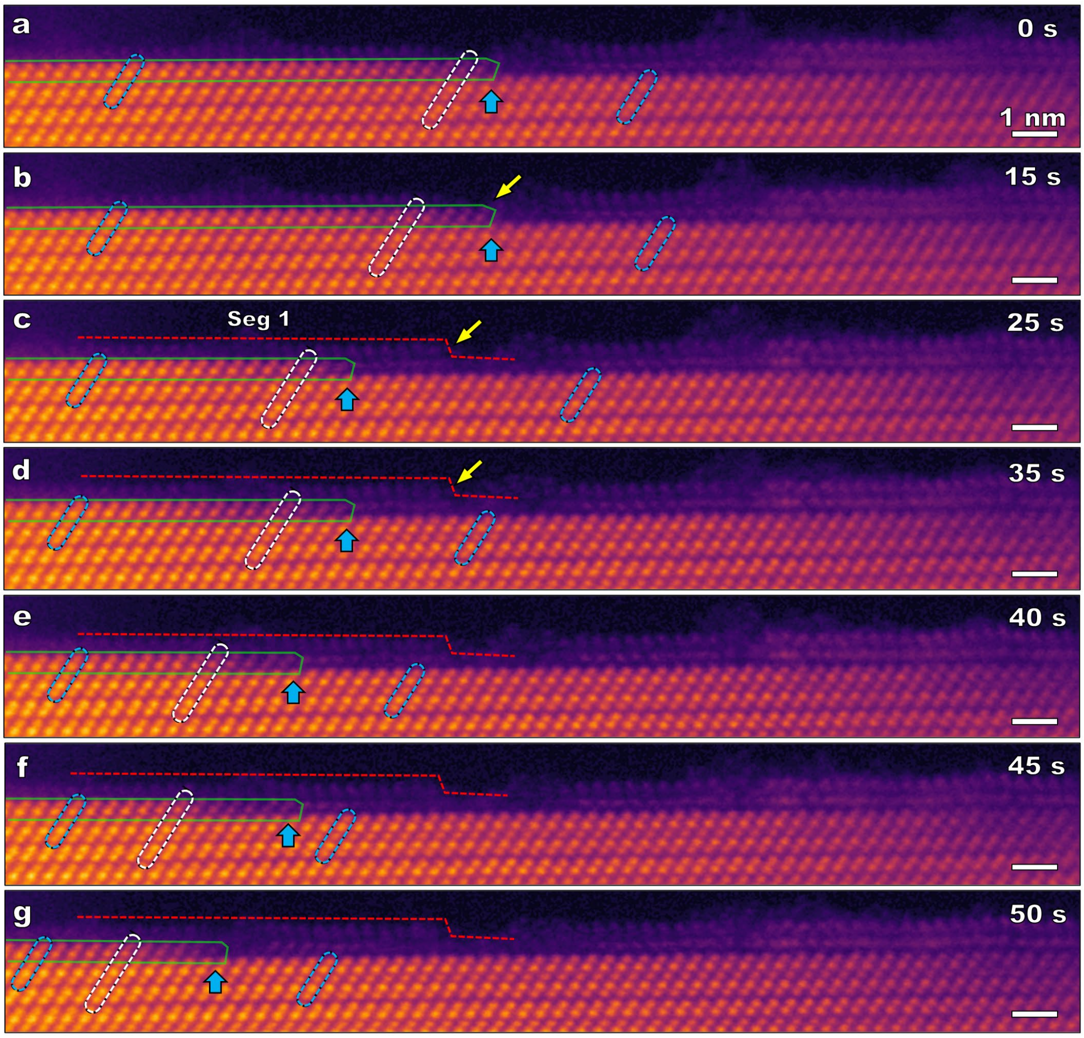

Supplementary Figure 20 | Additional example showing surface reconstruction process induced by cations diffusion. (a-g) Time-elapsed atomic resolution STEM images showing the cation diffusion and surface reconstruction. The cyan and white dotted rods mark the QLs and SLs, respectively. The cyan and yellow arrows indicate the dislocation cores and the steps at (0001) crystallography plane, respectively. The red dashed lines and green solid lines show the outline of the reconstructed surface the and the Ge/Bi extra half planes, respectively.

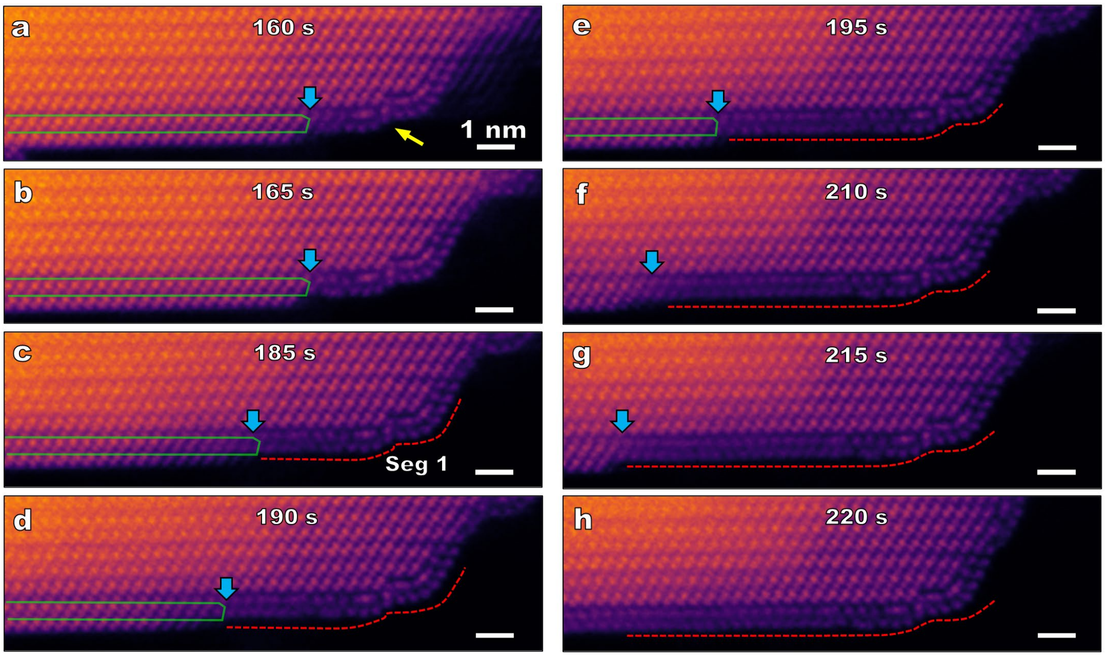

Supplementary Figure 21 | Additional example showing surface reconstruction process induced by cations diffusion. (a-h) Time-elapsed atomic resolution STEM images showing the cation diffusion and surface reconstruction. The cyan and yellow arrows and indicate the dislocation cores and the steps at (0001) surface, respectively. The red dashed lines and green solid lines indicate the outline of the reconstructed surface and the Ge/Bi extra half plane, respectively.

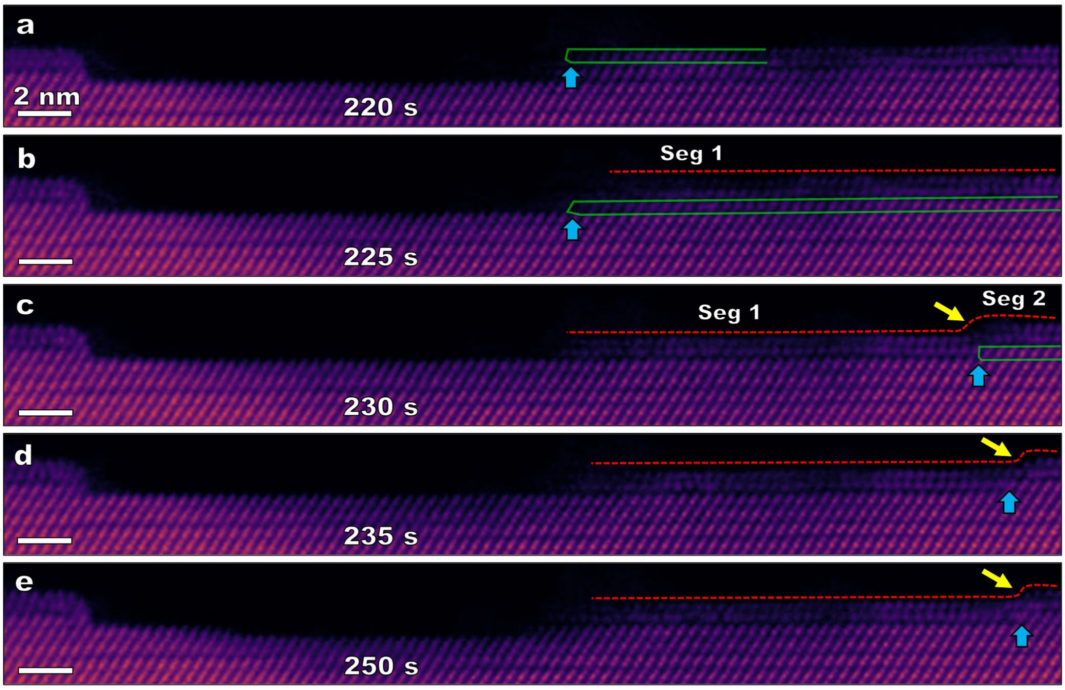

Supplementary Figure 22 | Additional example showing surface reconstruction process induced by cations diffusion. (a-e) Time-elapsed atomic resolution STEM images showing the cation diffusion and surface reconstruction. The cyan and yellow arrows and indicate the dislocation cores and the steps at (0001) surface, respectively. The red dashed lines and green solid lines indicate the outline of the reconstructed surface and the Ge/Bi extra half plane, respectively.

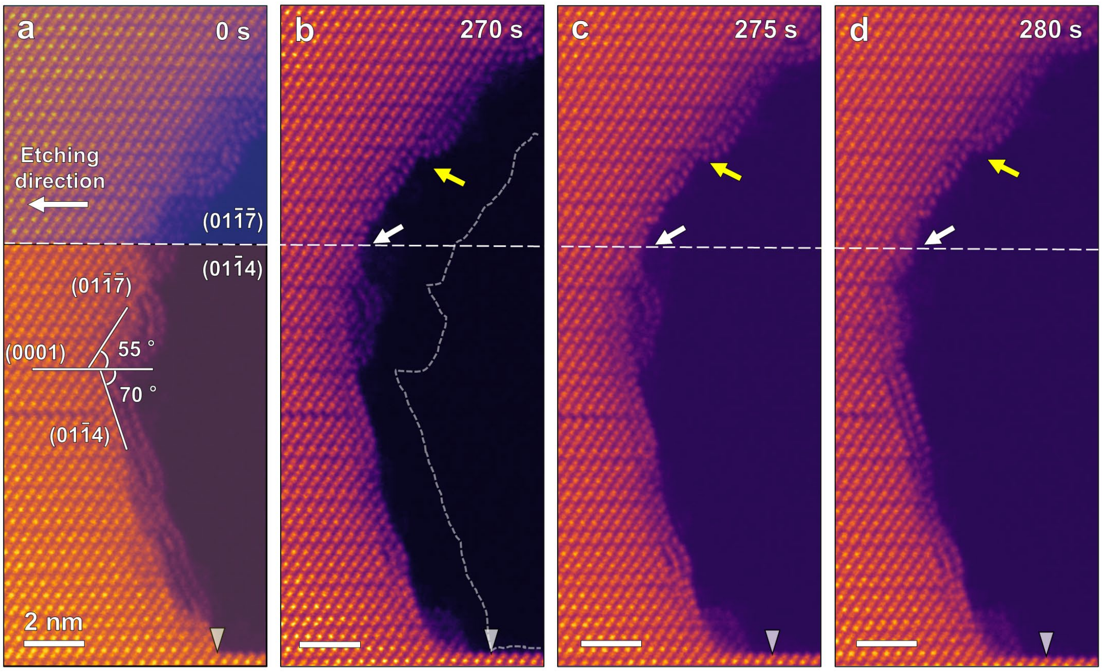

Supplementary Figure 23 | *In situ* STEM images acquired on the (01$\bar{1}\bar{7}$) and (01$\bar{1}$4) surfaces. (a-d) STEM images during (01$\bar{1}\bar{7}$) (upper part above the dotted line) and (01$\bar{1}$4) (lower part) surface reconstruction at 0 *s*, 270 *s*, 275 *s* and 280 *s*. Images are extracted from Supplementary Movie 4. The dotted curve at 270 *s* marks the initial surface at 0 *s*.

Supplementary Table 1 | Experimental and DFT *α* and *h* values of the projected octahedrons in GBT, GeTe and Bi_2Te_3 along the [$\bar{2}$110] zone axis.

Material	octahedron	Experimental data		DFT calculation	
		α (°)	*h* (pm)	*α* (°)	*h* (pm)
$GeBi_2Te_4$	Bi-centered Te_6	71	380	70	380
	Ge-centered Te_6	70	360	70	340
vdW gap (GBT)	Unoccupied Te_6	55	260	50	270
GeTe	Ge-centered Te_6	69	360	70	370
Bi_2Te_3	Bi-centered Te_6	71	380	70	380
vdW gap (Bi_2Te_3)	Unoccupied Te_6	55	260	50	270

Supplementary Table 2 | The diffusion energy barrier of Ge and Bi atom along the vdW gaps of Bi_2Te_3 and GBT layered structures.

Cation type	Diffusion energy barrier in the vdW gap (eV)	
	Bi_2Te_3	GBT
Ge	0.53	0.42
Bi	0.81	0.27

Supplementary Table 3 | The detail parameters (Cell size, KPOINTS, EDIFF and EDIFFG) of bulk GeTe, $GeTe_2$ TL, Ge_2Te_3 QL, Bi_2Te_3 and GBT structure for DFT calculations.

Structure	Calculated type	Cell size	KPOINTS	EDIFF	EDIFFG
Bulk GeTe	Structure relaxation	1×1×1	9×9×3	1E-6	-0.01
	DOS	1×1×1	15×15×3	1E-8	
$GeTe_2$ TL	Structure relaxation	1×1×1	9×9×1	1E-6	-0.01
	DOS	1×1×1	15×15×1	1E-8	
Ge_2Te_3 QL	Structure relaxation	1×1×1	9×9×1	1E-6	-0.01
Bi_2Te_3	NEB	3×2×1	2×3×1	1E-6	-0.03
	Surface energy			1E-5	-0.025
$GeBi_2Te_4$	Structure relaxation	2×6×1	3×1×1	1E-6	-0.02
	NEB	3×2×1	2×3×1	1E-6	-0.03
	Surface energy			1E-5	-0.025

Supplementary References

1. Karpinsky O., Shelimova L., Kretova M. & Fleurial J-P. X-Ray study of the *n*GeTe·*m*Bi_2Te_3 mixed layered tetradymite-like compounds. *J. Alloys. Compd*. **265**, 170-175. (1998).
2. Xiangyu M. et al. Ferroelectric engineering: Enhanced thermoelectric performance by local structural heterogeneity. *Sci. China Mater*. 1-8. (2022).
3. Okamoto K. et al. Observation of a highly spin-polarized topological surface state in $GeBi_2Te_4$. *Phys. Rev. B* **86**, 195304. (2012).
4. Sales D. et al. Distribution of bismuth atoms in epitaxial GaAsBi. *Appl. Phys. Lett.* **98**, 101902. (2011).
5. Zhang Q., Zhang L. Y., Jin C. H., Wang Y. M. & Lin F. CalAtom: A software for quantitatively analysing atomic columns in a transmission electron microscope image. *Ultramicroscopy* **202**, 114-120. (2019).
6. Hou F. et al. Te-vacancy-induced surface collapse and reconstruction in antiferromagnetic topological insulator $MnBi_2Te_4$. *ACS Nano* **14**, 11262-11272. (2020).
7. Lotnyk A. et al. Atomic structure and dynamic reconfiguration of layered defects invan der Waals layered Ge-Sb-Te based materials. *Acta Mater*. **141**, 92-96. (2017).
8. Wang J-J. et al. Genesis and effects of swapping bilayers in hexagonal $GeSb_2Te_4$. *Chem. Mater.* **30**, 4770-4777. (2018).
9. Henkelman G., Uberuaga B. P. & Jónsson H. A climbing image nudged elastic band method for finding saddle points and minimum energy paths. *J. Chem. Phys*. **113**, 9901-9904. (2000).
