# Supplementary material for: Direct observation of cation diffusion driven surface reconstruction at van der Waals gaps
Source: Nat Commun. 2023 Feb 2;14:554. doi: 10.1038/s41467-023-35972-9 (PMC9894939; doi:10.1038/s41467-023-35972-9)
Supplement: Supplementary file 4 — Description of Additional Supplementary Files [file 41467_2023_35972_MOESM4_ESM.pdf]

Description of Additional Supplementary Files:

Supplementary Movie 1: In situ low magnification TEM movie showing the morphology change of the $GeBi_2Te_4$ FIB sample heated from room temperature to 250 °C.

Supplementary Movie 2: In situ atomic resolution ADF-STEM movie showing Ge/Bi cation diffusion along a vdW gap.

Supplementary Movie 3: In situ atomic resolution ADF-STEM movie showing surface reconstruction and etching of (0001) surface.

Supplementary Movie 4: In situ atomic resolution ADF-STEM movie showing surface reconstruction and etching of $(01\bar{1}\bar{7})$ and $(01\bar{1}4)$ surfaces.

Supplementary Movie 5: In situ ADF-STEM movie showing pore expansion and anisotropic etching of a $GeBi_2Te_4$ FIB sample
